# A Practical Treatise on Inflammation of the Uterus and Its Appendages, and on Ulceration and Induration of the Neck of the Uterus

**Published:** 1850-05

**Authors:** 


Art. V.—*A Practical Treatise on Inflammation of the Uterus and its Appendages, and on Ulceration and Induration of the Neck of the Uterus.* By James Henry Bennet, M. D., M. R. C. P., Physician-Accoucheur to the Western General Dispensatory; formerly House-Physician (by concours) to the hospitals Saint Louis, Notre Dame, La Pitie, and La Salpetriere, Paris. Second American, from the second London edition. Philadelphia: Lea & Blanchard. 1850.

This is one of the most valuable guides in the treatment of the diseases of the Uterus and its appendages, that we know of. It is the production of a patient observer, and of a clear and accurate master of the science of diagnosis, in full possession of all the means for a perfect mastery of the subject. The former edition of the work was hailed with general approbation, by the medical profession; but the present edition is almost a new work.

The author claims a great deal of originality in various fields of investigation, and a careful study of the book, and an attentive observation at the bedside, will abundantly sustain his claims, and we earnestly commend this work to all who pay any attention to the diseases of females. It will be found eminently trustworthy.

The remarks of Dr. Bennet, on the use of the speculum in the treatment of the diseases of females, are amply confirmed by the admirable little treatise, written by Dr. Thomas R. Mitchell, of the Southeastern Lying-in Hospital, Dublin.

The admirable work of Dr. Bennet may be found at the bookstore of Beckwith & Morton.

